# Pentraxin 3 Levels in Young Women with and without Polycystic Ovary Syndrome (PCOS) in relation to the Nutritional Status and Systemic Inflammation

**DOI:** 10.1155/2020/1380176

**Published:** 2020-09-02

**Authors:** Katarzyna Wyskida, Grzegorz Franik, Piotr Choręza, Natalia Pohl, Leszek Markuszewski, Aleksander Owczarek, Paweł Madej, Jerzy Chudek, Magdalena Olszanecka-Glinianowicz

**Affiliations:** ^1^Health Promotion and Obesity Management Unit, Department of Pathophysiology, Medical Faculty in Katowice, The Medical University of Silesia, Katowice, Poland; ^2^Department of Gynecological Endocrinology, Medical Faculty in Katowice, The Medical University of Silesia, Katowice, Poland; ^3^Department of Statistics, Department of Instrumental Analysis, Faculty of Pharmaceutical Sciences in Sosnowiec, Medical University of Silesia, Katowice, Poland; ^4^Center Heart and Vascular Diseases, Internal and Metabolic Diseases, Mazovian Specialist Hospital in Radom, Radom, Poland; ^5^Pathophysiology Unit, Department of Pathophysiology, Medical Faculty in Katowice, The Medical University of Silesia, Katowice, Poland; ^6^Department of Internal Medicine and Oncological Chemotherapy, Medical Faculty in Katowice, The Medical University of Silesia, Katowice, Poland

## Abstract

**Objective:**

The aim of the study was to assess PTX3 levels in PCOS and non-PCOS women in relation to nutritional status and circulating markers of inflammation.

**Methods:**

The study enrolled 99 stable body mass PCOS women (17 normal weight, 21 overweight, and 61 obese) and 61 non-PCOS women (24 normal weight, 19 overweight, and 18 obese). Body composition was assessed by bioimpedance, and plasma levels of pentraxin 3 (PTX3), tumor necrosis factor-*α* (TNF-*α*), interleukin-6 (IL-6), and monocyte chemoattractant protein 1 (MCP-1) were measured. Homeostatic model assessment of insulin resistance (HOMA-IR) was made.

**Results:**

Plasma PTX3, TNF-*α*, and IL-6 levels and HOMA-IR were higher in PCOS than in non-PCOS group (*p* < 0.001). There were positive correlations between log_10_ (PTX3) and log_10_ (BMI), waist circumference and fat percentage, as well as log_10_ (HOMA-IR) and free androgen index but negative between log_10_ (estradiol) levels in PCOS. While in the non-PCOS group, the correlations between log_10_ (PTX3) and log_10_ (BMI), waist circumference and fat percentage, as well as log_10_ (HOMA-IR) were negative. The positive correlations between PTX3 and MPC-1 and log_10_ (IL-6) were shown in the PCOS group only. In multivariate regression analyses, variability in PTX3 levels in the PCOS group was proportional to log_10_ (BMI), waist circumference, and fat percentage, but inversely proportional to log_10_ (estradiol) levels. While in the non-PCOS group, PTX3 levels were inversely proportional to all anthropometric parameters.

**Conclusions:**

Our results show that the decrease in PTX3 levels observed in obese is distorted in PCOS by microinflammation, and possibly, dysfunction of stroma adipose tissue and liver steatosis is reflected by enhanced insulin resistance.

## 1. Introduction

The acute phase proteins named pentraxins are involved in the cascade of inflammatory responses that belongs to glycoproteins constructed of five units. The group of short pentraxins including C-reactive protein (CRP) and serum amyloid A (SAA) is synthesized mostly in the liver, and their synthesis is stimulated by IL-6. The group of long pentraxins includes PTX3 expressed locally in the site of inflammation [1, 2]. PTX3 is secreted by numerous types of cells including monocytes, macrophages, dendric cells, endothelial cells, hepatocytes, smooth muscle cells, and fibroblasts [3] after stimulation by interleukin-1*β* (IL-1*β*), TNF-*α*, interleukin-10 (IL-10), and toll-like receptor (TLR) agonists as well as oxidized low-density lipoproteins (oxLDL), but not by IL-6 [4]. In adipose tissue, PTX3 is expressed in the stromal-vascular cells, but not in adipocytes. However, this expression in adipocytes can be reinduced during TNF-*α* exposure [5].

The previously published studies showed that systemic microinflammation assessed by circulating TNF-*α* and IL-6 levels in PCOS women is associated with nutritional status and not with PCOS occurrence *per se* [6]. However, some studies found higher circulating CRP levels in PCOS women [7]. In addition, a meta-analysis confirms observations that IL-6 and TNF-*α* levels are related to obesity but not PCOS occurrence, whereas CRP levels are elevated in PCOS independent of obesity [8]. However, the results of studies assessed circulating PTX3 levels in PCOS women are inconsistent [9–13], and its associations with nutritional status and insulin resistance are unclear [14–16]. So far, only one study assessed the association between PTX3 and TNF-*α* and IL-6 levels. This study revealed a positive correlation between these parameters in morbidly obese subjects [17]. Therefore, the aim of the study was to assess PTX3 levels in PCOS and non-PCOS women in relation to nutritional status and circulating inflammation markers.

## 2. Methods

### 2.1. Study Population

The cross-sectional study involved 99 PCOS women (17 normal weight, 21 overweight, and 61 obese), inpatients of the Department of Endocrinology Gynecology from 2015 to 2018 and 61 non-PCOS women (24 normal weight, 19 overweight, and 18 obese) referred to Obesity Management Outpatients Clinic from 2015 to 2018 or voluntaries referred to Gynecology Outpatients Clinic for first-time contraceptive advice (normal weight) with stable body mass during last 3-month period. The diagnosis of PCOS was based on Rotterdam ESHRE/ASRM criteria from 2003 [18]. Patients diagnosed with type 1 and 2 diabetes, hypertension, dyslipidemia, any cardiovascular disease, thyroid dysfunctions, Cushing's syndrome, androgen-secreting tumour, and enzyme deficiency (21-hydroxylase in particular), and decreased ovary reserves were not enrolled. Any pharmacological therapy and smoking and alcohol abuse were among the exclusion criteria. The study protocol was approved by the Ethical Committee of the Medical University of Silesia. Each participant signed informed consent.

All the study women were tested within 3 and 5 days of the menstrual cycle. Anthropometric measurements (body mass and height) were performed, and BMI was calculated according to the standard formula. Body composition was assessed by bioimpedance method using Bodystat 1500 (Douglas, Isle of Man). 15 ml samples of venous blood were withdrawn in the morning between 8.00 and 9.00 a.m., after an overnight fast (16 h). The blood samples were collected according to the recommendation of the kits' manufacturers. Serum and plasma samples were stored frozen in −70°C.

### 2.2. Biochemical Measurements

Plasma PTX3 levels were determined by the ELISA method (BioVendor, Brno, The Czech Republic) with the limit of quantification (LOQ) of 0.08 ng/mL and intra-and interassay coefficients of variations of 6% and 12%, respectively. The ELISA method was also used to determine concentrations of TNF-*α*, IL-6, and MCP-1 (R&D Systems, Michigan, USA) with an LOQ of 16 pg/mL, 3 pg/mL, and 31 pg/mL, respectively; intra-and interassay coefficients of variations were 8.7% and 10.4% for TNF-*α*; 7.8% and 9.6% for IL-6; and 4.2% and 5.9% for MCP-1.

In addition, plasma glucose and lipids were estimated by colorimetric methods using the commercially available test kits (Roche, Switzerland). Serum insulin concentration was determined by the electrochemiluminescence immunoassay (ECLIA) method using the Cobas E411 analyzer (Roche Diagnostics GmbH, Mannheim, Germany) with a limit of quantification (LOQ) of 0.2 *µ*IU/ml and intra- and interassay coefficients of variations of 2.0% and 2.8%, respectively. Insulin resistance was assessed on the basis of HOMA-IR calculated using standard formula.

Serum estradiol (E2), testosterone, and sex hormone binding globulin (SHBG) were determined by ECLIA method using the Cobas E411 analyzer (Roche Diagnostics GmbH, Mannheim, Germany) with LOQs: 5.0 pg/mL, 0.025 ng/mL, and 0.35 nmol/L, respectively; the respective intra-and interassay coefficients of variations were 4.6% and 9.9% for E2, 4.7% and 8.4% for testosterone, and 2.7% and 5.6% for SHBG. In addition, the free androgens index (FAI) was calculated using the standard formula.

### 2.3. Data Analysis

The cutoff HOMA-IR value of 2.5 and over was used to the defined insulin resistance [19]. Nutritional status was scored according to WHO criteria [20].

### 2.4. Statistical Analysis

Statistical analysis was performed with STATISTICA 12.0 software (TIBCO Software Inc., Palo Alto, USA). Nominal and ordinal data were expressed as percentages, while interval data were expressed as mean value ± standard deviation in case of normal distribution or as median with lower and upper quartile in case of data with skewed or non-normal distribution. Distribution of variables was evaluated by the Shapiro–Wilk test and a quantile-quantile (Q-Q) plot, and homogeneity of variances was assessed by the Fisher–Snedeckore test. For comparison of data between group with and without PCOS, the Student's *t*-test for independent data (in case of normal data distribution or after logarithmic normalization, if appropriate, in case of skewed distribution) or the nonparametric Mann–Whitney *U*-test (in non-normal data distribution) was used. The Pearson correlation coefficient was used as a measure of association between analyzed variables. Results in group with and without PCOS were shown with a correlogram plot. Multivariable stepwise backward regression analysis was performed for PTX3 serum levels as an independent variable with potentially explanatory variables: body mass index (BMI) (model I), fat percentage (model II), waist (model III), and age, serum levels of SHBG, IL-6, TNF-*α*, estradiol and 17-OH-progesterone, MCP-1, FAI, and HOMA-IR. The influence of factors was assessed based on the standardized regression coefficients. The Cook–Weisberg test was used to test heteroskedasticity and the Remsey RESET test was used to test the linearity of regression. The variance inflation factor VIF was calculated to check the multicollinearity occurrence. The goodness of fit of obtained regression models was assessed with the adjusted determination coefficient *R*^2^. The association between PTX3 levels with body mass index in group with and without PCOS was presented with the distance-weighted least squares method and shown as plots. All tests were two-tailed. The results were considered as statistically significant with a *p* value of less than 0.05.

## 3. Results

The PCOS group was characterized by significantly higher BMI values, fat mass percentage and waist circumference, as well as insulin levels and HOMA-IR values than the group without PCOS. As expected, PCOS women were found with higher FAI values and lower estradiol and SHBG levels. Furthermore, higher plasma PTX3, TNF-*α*, and IL-6 levels in PCOS than non-PCOS groups were shown. The characteristics of study groups and subgroups are presented in Table 1.

**Figure 1:**
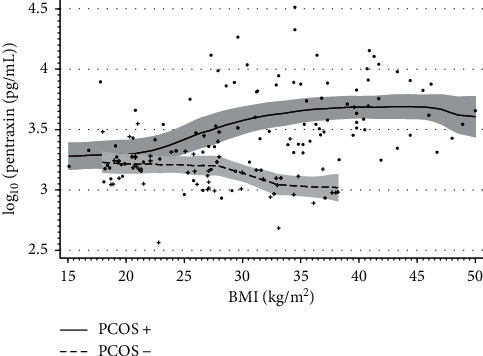
The relationship between PTX serum levels and BMI in PCOS and non-PCOS groups. Gray areas depict 95% confidence intervals.

**Figure 2:**
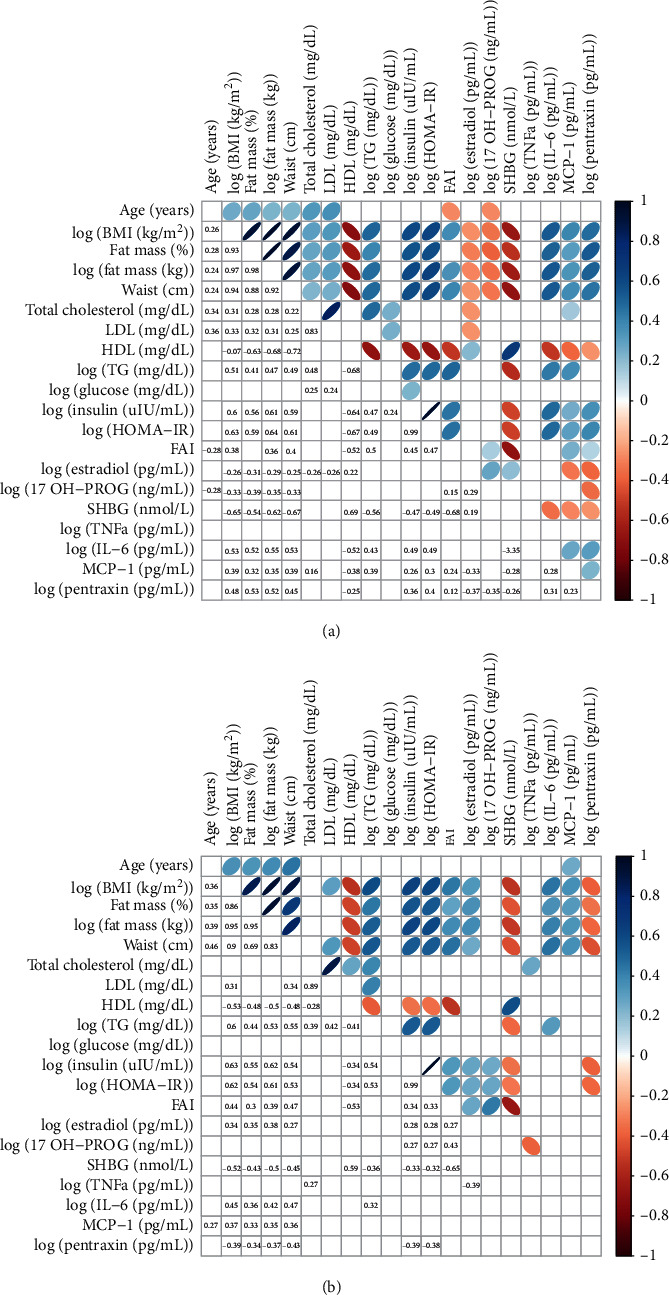
Correlogram of analyzed variables in groups with and without PCOS.

The opposite correlation between log_10_ (BMI) and log_10_ (PTX3) was shown. This correlation was positive in the PCOS group, while it was negative in the non-PCOS group(Figure 1). Notwithstanding, in both study groups, the correlations between log_10_ (BMI) values, waist circumference, as well as fat percentage and levels of MCP-1 and log_10_ (IL-6), but not log_10_ (TNF-*α*), were positive (Figure 2). In addition, in the PCOS group, positive correlations between log_10_ (PTX3) and waist circumference and fat percentage as well as log_10_(Insulin) levels, log_10_ (HOMA-IR), and FAI values and negative correlations between log_10_ (Estradiol) levels were observed. While in the group without PCOS, negative correlations between log_10_ (PTX3) and waist circumference and fat percentage as well as log_10_ (Insulin) levels and log_10_ (HOMA-IR) were found. Circulating levels of PTX3 were proportional to MPC-1 and IL-6 in the PCOS group only (Figure 2).

A multivariable stepwise backward linear regression model for log_10_ (PTX3) as the independent variable, with explanatory variables such as log_10_ (BMI) values or waist circumference or fat percentage and plasma levels of log_10_ (TNF-alpha), log_10_ (IL-6), MCP-1, log_10_ (estradiol), SHBG levels, FAI, and log_10_ (HOMA-IR) values, showed that all anthropometric parameters (BMI values, waist circumference, and fat percentage) were positively affecting plasma PTX3 levels while log_10_ (Estradiol) levels were inversely affecting plasma PTX3 levels in the PCOS group. While in the group without PCOS, log_10_ (PTX3) levels were inversely related by all anthropometric parameters only. The best model explaining PTX3 variability in the PCOS group was the model including fat percentage and in the non-PCOS group, model including waist circumference. In the combined analysis (with and without PCOS), the occurrence of PCOS was the most important factor explaining the variability of log_10_ (PTX3), and the best model enclosed body fat percentage as the fat depot measure (Table 2).

## 4. Discussion

To the best our knowledge, this is the first study that assessed the effect of nutritional status, insulin resistance, hormonal status, and inflammation on circulating PTX3 levels in PCOS with reference to non-PCOS women. We showed similar PTX3 levels in normal-weight PCOS and non-PCOS women, but remarkably higher in obese PCOS women. In addition, we found an opposite association between fat mass percentage and PTX3 levels in PCOS (positive) than non-PCOS (negative) and an inverse relationship with estradiol level in PCOS only.

These results concerning increased PTX3 levels in PCOS women are contradictory to those obtained by Sahin et al. [11] and Tosi et al. [13], that showed lower PTX3 levels in PCOS than in the control group and the observation of Sari et al. [12] that revealed no difference between women with and without PCOS, while these are in accordance with results reported by Aydogdu et al. [9]. These differences partially may be a result of used ELISA kits, produced by different manufactures. It should be stressed that any of commercial ELISA kits for PTX3 was validated against the quantitative western-blot method.

As it was mentioned before, our study showed a positive correlation between circulating PTX3 levels and BMI values, waist circumference, and fat percentage in the PCOS group, while the correlation was negative in the non-PCOS group. These results were confirmed by multivariable stepwise backward regression analysis. The positive correlation between PTX3 levels and BMI as well as visceral obesity in PCOS women was also observed by Aydogdu et al. [9]. However, Sahin et al. [11] found the inverse association between PTX3 levels and BMI values, typical for those unaffected by PCOS cohorts. However, our results suggested that the incompatibilities between our and previously published studies may be more complex. Our PCOS group compromised a subset of subjects with much higher BMI values, waist circumference and fat percentage, and HOMA-IR values in comparison with the non-PCOS group. These results are contrary to those described previously in the group of women newly diagnosed with PCOS that revealed inverse relation between PTX3 and insulin resistance [11] and in accordance with others [9]. The negative correlation between anthropometric measurements and PTX3 revealed in non-POCS suggests that adipose tissue is not a major source of PTX3, and that insulin resistance inhibits its production, and the severity of inflammation is too small to induce PTX3 expression not only in adipocytes. It was recently shown that PTX3 gene expression is upregulated in the visceral fat depot of obese subjects but without significant effect on its concentration in the circulation [21]. Unexpectedly, the increasing degree of obesity, insulin resistance, and inflammation (reflected by MCP-1 and IL-6 levels) in PCOS was associated with increased PTX3 production. Our previous study revealed secondary to insulin resistance and hyperandrogenism impairment of hormonal stroma adipose tissue function in PCOS independent of nutritional status [22]. Thus, we suppose that the increased PTX3 levels and its correlation with anthropometric parameters in PCOS women are the effect of stroma adipose tissue dysfunction. The second potential explanation may be an increased PTX3 production by hepatocytes in the fatty liver. This hypothesis is supported by the observed correlation between PTX3 levels and insulin levels, HOMA-IR values, and IL-6 levels found in our study. In addition, the positive correlation between plasma PTX3 levels with NAFLD activity score, fibrosis stage, and steatosis grade has been described in adults [23]. Moreover, it has also been shown that, in adolescents, PTX3 levels increased progressively with the severity of the fatty liver [24]. It should also be noted that meta-analysis of 17 studies including 2715 women diagnosed with PCOS and NAFLD and 2619 with NAFL revealed that risk of NAFLD development is higher in PCOS, and this risk is independent of obesity (diagnosed based on BMI) and may be a result of hyperandrogenism [25]. Our study showed a positive correlation between PTX3 levels and FAI values in the PCOS group. However, to fully confirm these hypotheses, imaging studies with an assessment of liver steatosis in magnetic resonance (MR) and PTX3 levels in women with PCOS are necessary. It cannot be ruled out that additional sources of increased circulating PTX3 levels in women with PCOS are cells of the immune system, as indicated by the relationship between PTX3 and MCP-1 levels observed in our study, as well as higher monocytes count in PCOS than in non-PCOS women [26]. Further studies are necessary to explain associations between PTX3 production and hormonal dysfunction of adipose tissue as well as liver steatosis and fibrosis. The role of PTX3 levels in metabolic and cardiovascular diseases development in PCOS should also be examined. The role of elevated PTX3 levels as a cardiovascular risk factor was confirmed in the Multi-Ethnic Study of Atherosclerosis including 2838 subjects [27]. Besides, our previously published study showed that circulating PTX3 levels may be the marker of endothelial dysfunction (the early stage of atherosclerosis development) dependent on nutritional status in young PCOS women without cardiovascular diseases [28]

The main limitation of the present study is small sample size; however, in comparison with previous studies, assessing the role of PTX3 in PCOS our study group was the biggest. The second limitation is also the assessment of body composition on the basis of bioimpedance method and not DXA method; therefore, the assessment of visceral fat depot was possible only indirectly based on the waist circumference measurement. The other limitations were the lack of assessment of liver steatosis with magnetic resonance (MR) and hormonal function of adipose tissue on the basis of circulating adipokine levels. Also, a highly selected cohort of PCOS women without obesity-related diseases, e.g., hypertension, diabetes, and lipid disorders may be considered as a limitation. However, such restricted inclusion criteria made the group much more homogenous and enabled an analysis of the relationship between nutritional status as the potential confounder. As a consequence, we cannot exclude that obesity-related comorbidity may increase PTX3 levels, as it was shown in patients with coronary artery disease [29]. Nevertheless, it should be noted that our study is the first assessed complex association between PTX3 levels and nutritional status, insulin resistance, hormone levels, and systemic microinflammation in young PCOS and non-PCOS women.

## 5. Conclusions

Our results show that the decrease in PTX3 levels observed in obese women is distorted in PCOS by microinflammation, and possibly, dysfunction of stroma adipose tissue and liver steatosis is reflected by enhanced insulin resistance.

## Figures and Tables

**Table 1:** Baseline characteristics and comparison between PCOS and non-PCOS group.

	PCOS (*N* = 99)	Non-PCOS (*N* = 60)	*p*
Age (years)	26.9 ± 5.8	26.6 ± 5.0	0.72
Body mass (kg)	90.7 ± 25.9	73.9 ± 15.7	<0.001
BMI (kg/m^2^)	34.4 (27.1–39.8)	26.7 (21.2–32.1)	<0.001
Fat mass percentage	42.8 ± 10.0	36.1 ± 9.0	<0.001
Fat mass (kg)	43.6 (27.0–52.7)	27.1 (15.5–37.2)	<0.001
Waist circumference (cm)	96.5 ± 19.8	86.2 ± 14.3	<0.001
Total cholesterol (mg/dL)	186.3 ± 30.6	175.6 ± 33.5	<0.05
LDL cholesterol (mg/dL)	98.1 ± 25.4	93.4 ± 27.7	0.86
HDL cholesterol (mg/dL)	39.2 ± 14.5	51.1 ± 15.9	<0.001
Triglycerides (mg/dL)	115.0 (85.0–181.0)	69.0 (56.0–105.0)	<0.001
Glucose (mg/dL)	91.0 (84.0–97.0)	90.0 (84.0–97.5)	0.90
Insulin (*μ*IU/ml)	16.59 (10.48–25.51)	7.23 (4.66–9.57)	<0.001
HOMA-IR	3.63 (2.34–5.67)	1.61 (0.98–2.25)	<0.001
FAI	1.06 (0.43–1.91)	0.37 (0.19–0.81)	<0.001
Estradiol (pg/mL)	98.7 (64.1–195.7)	234.0 (162.5–344.0)	<0.001
17-OH-progesterone (ng/mL)	1.27 (0.85–1.80)	1.51 (1.08–2.05)	0.06
SHBG (nmol/L)	45.7 ± 30.6	80.3 ± 49.0	<0.001
TNF-*α* (pg/mL)	0.97 (0.57–1.85)	0.72 (0.56–0.99)	<0.001
IL-6 (pg/mL)	1.61 (0.82–2.89)	0.73 (0.45–1.28)	<0.001
MCP-1 (pg/mL)	272.0 ± 151.6	245.0 ± 102.8	0.18
PTX3 (ng/mL)	3.4 (2.1–6.9)	1.4 (1.1–1.9)	<0.001

**Table 2:** Results of multivariable stepwise backward regression factors influencing PTX3 serum levels in subjects with and without PCOS.

log_10_ (pentraxin (pg/mL))	PCOS			Non-PCOS			All		
	*β* (SE (*β*))			*β* (SE (*β*))			*β* (SE (*β*))		
	Model I	Model II	Model III	Model I	Model II	Model III	Model I	Model II	Model III
PCOS +	—	—	—	—	—	—	0.248 (0.050)^#^	0.248 (0.049)^#^	0.287 (0.050)^#^
log_10_ (BMI) (kg/m^2^)	0.948 (0.234)^#^	—	—	−0.789 (0.242)^*∗∗*^	—	—	0.586 (0.182)^*∗∗*^	—	—
Fat percentage (%)	—	0.142 (0.030)^#^	—	—	−0.007 (0.003)^*∗∗*^	—	—	0.008 (0.002)^#^	—
Waist circumference (cm)	—	—	0.005 (0.002)^*∗∗*^	—	—	−0.006 (0.002)^#^	—	—	—
log_10_ (estradiol (pg/mL))	−0.237 (0.082)^*∗∗*^	−0.211 (0.081)^*∗*^	−0.258 (0.084)^*∗∗*^	—	—	—	−0.240 (0.066)^#^	−0.237 (0.065)^#^	−0.262 (0.067)^#^
Adjusted *R*^2^	0.244	0.280	0.202	0.140	0.097	0.172	0.395	0.403	0.353

^*∗*^ *p* < 0.05;  ^*∗∗*^*p* < 0.01;  ^#^*p* < 0.001.

## Data Availability

The data used to support the findings of this study are available from the corresponding author upon request.

## Abbreviations

BMI: Body mass index
DXA: Dual-energy X-ray absorptiometry
ELISA: Enzyme-linked immunosorbent assay
ECLIA: Electrochemiluminescence assay
HOMA-IR: Homeostatic model assessment insulin resistance
MCP-1: Monocyte chemoattractant protein-1
MR: Magnetic resonance
NAFLD: Nonalcoholic fatty liver disease
NASH: Nonalcoholic steatohepatitis
PCOS: Polycystic ovary syndrome
PTX3: Pentraxin 3
TNF-*α*: Tumor necrosis factor-*α*.
